# Case study: Clinical research

**Published:** 2023-01-30

**Authors:** Allen Foster

**Affiliations:** Professor of International Eye Health: International Eye Health Centre, London School of Hygiene & Tropical Medicine, London, UK.

## Abstract

While working as an ophthalmologist in a hospital in Tanzania in the 1980s, I became interested in why so many children were blind due to corneal scarring. Keratomalacia (drying and clouding of the cornea) due to vitamin A deficiency had been reported in Asia, but Bitot's spots – normally associated with keratomalacia – were rarely seen in African children. The children's parents often said the eye problems and blindness was due to measles. In addition, herpes simplex keratitis had been reported as a cause of corneal ulceration after measles in Nigeria.

By good fortune, I met Prof Al Sommer, who was working on vitamin A deficiency in Indonesia and Nepal. With his advice and encouragement, we started a prospective study to investigate and photograph all cases of corneal ulceration in children who came to the hospital where I was working. Over three years, we documented 130 cases of corneal ulceration in children and found that, although herpes simplex virus was the commonest cause of ulceration overall, vitamin A deficiency was the major cause of bilateral ulceration, subsequent blindness, and mortality in this series of patients.

As so often happens, our research led to more questions and further studies, including one which showed that vitamin A supplementation reduced mortality in children hospitalised with measles. This work contributed to the evidence that led WHO and UNICEF in 1997 to announce a programme of vitamin A supplementation for children with measles.

**Figure:**
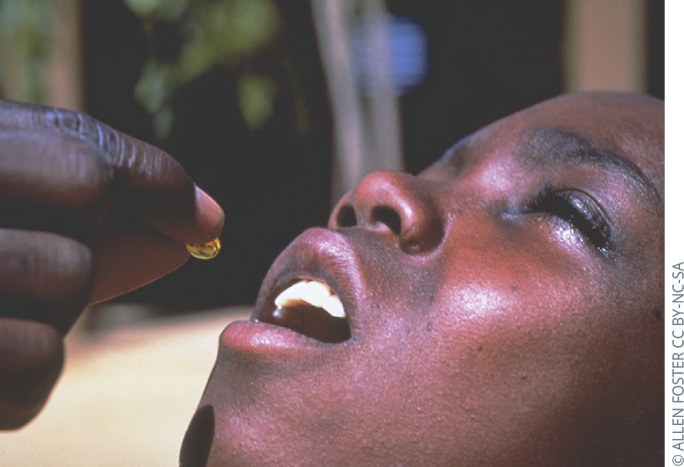
Giving vitamin A to a child. tanzania

What did I learn from this initial research experience?

1. Identify a clear research question
2. Take time to plan the study
3. Work with colleagues who have other expertise.
